# The Importance of Thermodynamic Data for Pyrrole Pyrolysis and Combustion Chemistry

**DOI:** 10.1002/cphc.70563

**Published:** 2026-09-20

**Authors:** Atmadeep Bhattacharya, Kari Laasonen, Ossi Kaario, Alexander A. Konnov

**Affiliations:** ^1^ Department of Energy and Mechanical Engineering Aalto University School of Engineering Espoo Finland; ^2^ Department of Chemistry and Material Science Aalto University School of Chemical Engineering Espoo Finland; ^3^ Division of Combustion Physics Lund University Lund Sweden

**Keywords:** ab initio calculations, combustion, pyrolysis, pyrrole, thermodynamics

## Abstract

In this work, the CCSD(T)/Extrapolate(3/4,def2)//PBE0/def2‐QZVP levels of theory were utilized in ORCA 6.1.1 to derive enthalpy and entropy of formation at 298.15 K for 32 species that include both radicals and stable molecules relevant to the pyrolysis and combustion of pyrrole. The comparison revealed major discrepancies in thermochemical data of species like allyloxy cyanide, *cis*‐formimidoyl, cyanomethylidyne, cyano‐propen‐2yl radical, cyano‐propen‐4yl radical, carbonyl cyanide, and propionitrile primary radical. The effects of the thermochemical data computed in this work on the performance of Pelucchi et al. (Energy & Fuels, 2021) and Konnov et al. (Proceedings of the Combustion Institute, 2024) mechanisms were assessed. It was found that the updated thermochemical data improved the mole fraction predictions of certain species (e.g., hydrogen cyanide and acetylene) during the pyrolysis of pyrrole. However, an opposite trend of predictive performance deterioration was observed for species like acetonitrile during both pyrolysis and combustion (φ = 0.5, 1, and 2) of pyrrole. In some cases, one model’s performance improved, whereas the other’s deteriorated. Therefore, this study reveals the significance of simultaneously improving the accuracy of both thermodynamic and reaction parameters of future pyrrole combustion chemistry models.

## Graphical abstract

High level ab initio quantum chemical computations with ORCA 6.1.1 are done to derive enthalpy and entropy of formation at 298.15 K for 32 species that include both radicals and stable molecules relevant to the pyrolysis and combustion of pyrrole. The effects of thermochemical data on chemical model performance are assessed. This work reveals the importance of thermochemistry for pyrrole combustion prediction.

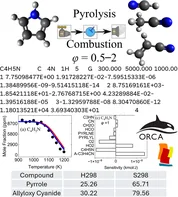

## 1 Introduction

Nonlignocellulosic biomass like algae, manure, food waste, and sewage sludge produce various N‐species upon being combusted, gasified, or pyrolyzed [1]. Pyrrole ($C_{4}H_{5}N$) is a key species in the fuel‐N reaction mechanism because it is the smallest molecule with an N atom in the heterocycle ring [2]. In an early effort to understand $C_{4}H_{5}N$ chemistry, Patterson et al. [3] investigated its pyrolysis and identified products like methane, ethylene, acetylene, benzene, hydrogen cyanide, and ammonia. Later, chemical kinetic models were proposed and validated against $C_{4}H_{5}N$ pyrolysis data from a shock tube [4, 5]. Based on the work of Mackie et al. [5], Lumbreras et al. [6] proposed a combustion mechanism of $C_{4}H_{5}N$ for the first time in the literature.

The combustion chemistry of $C_{4}H_{5}N$ has been investigated by multiple groups in the last couple of decades. Tian et al. [7] studied low pressure premixed laminar $C_{4}H_{5}N$–$O_{2}$ flames with tunable synchrotron photoionization and molecular beam mass spectrometry techniques. Many key combustion intermediates of $C_{4}H_{5}N$ oxidation, like nitrogen ($N_{2}$), nitric oxide (NO), nitrogen dioxide (${\text{NO}}_{2}$), isocyanic acid (HNCO), and 2‐propenenitrile (${\text{CH}}_{2}\text{CHCN}$) were detected in this work [7]. MacNamara and Simmie [8] measured ignition delay times of $C_{4}H_{5}N$–$O_{2}$ mixtures in a shock tube. Yamamoto et al. [9] proposed a detailed kinetic model consisting of 89 species and 505 reactions for $C_{4}H_{5}N$–$O_{2}$–$H_{2}O$–$N_{2}$ mixtures. The model [9] was validated against experimental data from a flow reactor designed for obtaining plug flow conditions.

The combustion mechanism of $C_{4}H_{5}N$ is still under investigation. Luo et al. [10] measured the ignition delay times of $C_{4}H_{5}N$ in $O_{2}$/${\text{CO}}_{2}$ atmospheres in a shock tube. The HUST pyrrole model with updated reaction rate parameters for $C_{4}H_{5}N$ together with allene, propyne, and propargyl radicals was proposed in this work [10]. In another work, Wu et al. [11] studied the pyrolysis of $C_{4}H_{5}N$ using a jet‐stirred reactor (JSR). The experimental data [11] were used to validate a detailed chemical kinetic model with 196 species and 2939 reactions. It was observed in this work [11] that nitriles like methyl isocyanide, cyanogen, isocyanogen, propionitrile, propanedinitrile, and butanedinitrile are the main N carriers during $C_{4}H_{5}N$ pyrolysis. Pelucchi et al. [12] investigated $C_{4}H_{5}N$ pyrolysis and combustion chemistry with a chemical kinetic model validated against experimental data from a JSR at atmospheric pressure. Lubrano Lavadera et al. [2] measured the laminar burning velocity of $C_{4}H_{5}N$ using a heat flux burner. For chemical kinetic modeling, reactions of $C_{4}H_{5}N$ were compiled and modified from the literature in this work [2].

Recently, Chen et al. [13] published a revised chemical kinetic model where reaction rate coefficients of H atom abstraction reactions from $C_{4}H_{5}N$ by OH, H, ${\text{CH}}_{3}$, and ${\text{HO}}_{2}$ were updated using theoretical calculations. In this model [13], $C_{4}H_{5}N$ + OH addition reactions were also implemented. Additionally, the thermodynamic properties of newly introduced species in this work [13] were evaluated at CCSD(T)/CBS/M06‐2x/6‐311 + G(d,p) level of theory. In another recent work, Chen et al. [14] investigated the formation of N‐containing pollutant species in counterflow diffusion flames of $C_{4}H_{5}N$. In this work [14], the chemical kinetic model from an earlier work [13] was updated with reaction rate parameters from the literature. Similarly, Wu et al. [15] proposed an updated pyrrole/pyridine model consisting of 233 species and 1569 reactions, including pyridine and pyrrole interaction. The thermodynamic parameters related to pyrrole chemistry in this model [15] were directly imported from Pelucchi et al. [12] Recently, Goldsborough et al. [16] proposed a detailed chemical kinetic model of pyrrolidine where the pyrrole subset from Pelucchi et al. [12] and Chen et al. [13] was implemented. Furthermore, the thermodynamic properties of pyrrolidine and its fuel radicals were updated in this work [16] using ab initio calculations.

In chemical kinetic modeling of combustion, thermodynamic data are as important as reaction rates. The thermochemical dataset is used for the calculation of reaction equilibrium constants. These equilibrium constants are employed in the determination of the reverse rate coefficients of a chemical reaction, where the forward rate coefficients are specified. Additionally, the temperature during a combustion simulation is computed from the heat release of the reactions and the specific heat of the gas mixture. Accurate estimation of the system temperature is very important since the elementary reactions have temperature‐dependent rates. The importance of thermochemical data was pointed out by Martin and Brown [17] in the early days of combustion chemistry research. An overview of computational thermodynamic data evaluation can be found in the recent work of Green [18] and Wu et al. [19]. While the kinetic rate parameters are usually considered for improving the performance of chemical mechanisms, fewer efforts have been made towards exploring the importance of thermochemical parameters during model development [20].

Turányi et al. [21] and Zádor et al. [22] reported the sensitivity of methane combustion to the thermodynamic parameters in the Leeds methane oxidation mechanism [23]. It was found that the maximum adiabatic flame temperature and maximum concentrations of H, O, and OH radicals are strongly influenced by the heat of formation data [21]. Although the contributions of the thermodynamic parameters to the total uncertainty are mostly lower than those of the kinetic ones, stoichiometric methane‐air flames show a high share of thermodynamic uncertainty for parameters like maximum flame temperature and maximal concentration of OH radical [22]. Recently, vom Lehn et al. [24] reported that the ignition delay time of diethyl ether may be highly sensitive to the enthalpy of formation of certain species. In another work, vom Lehn et al. [25] showed that the highest uncertainty arising from thermochemical parameters in ignition delay time prediction lies in the negative temperature coefficient (NTC) regime. Furthermore, vom Lehn et al. [26] also investigated the influence of thermochemical parameters on chemical kinetic model predictions for 2‐methylhexane ignition. It was observed that the highest sensitivities were present in the NTC regime for different groups pertaining to the peroxy and hydroperoxide moieties in the intermediate species [26].

Accurate experimental evaluation of thermochemical properties is impractical for a large number of species [20]. Therefore, the group additivity method [27, 28] and ab initio quantum chemical calculations [29] are commonly used in this context. Estimations of thermodynamic data with the group additivity method and ab initio calculations have been implemented in many state‐of‐the‐art automatic tools [30, 31] as well. Ghosh et al. [32] used heat of formation values calculated with ab initio methods by Elliott et al. [33] for a set of 192 species, including alkanes, alkyl radicals, alkyl hydroperoxides, alkyl‐peroxy radicals, and hydroperoxy‐alkyl radicals, to derive new group additivity values. In a recent work by Langer et al. [34], it was shown that the ${\mathrm{NO}}_{x}$ formation in low‐pressure methane–O_2_–N_2_ flames is highly sensitive to the specific heats of N_2_, NO, N, and NCN. On the other hand, the heat capacities of N_2_H_2_, N_2_H_3_, and NH were shown to have a significant influence on the laminar burning velocities of ammonia/air mixtures [34].

In a recent work by Breuer et al. [35], the effects of changing the thermodynamic parameters on the ignition delay time predictions of 2‐, 3‐, 4‐methylanisole isomers were clearly demonstrated. Furthermore, as seen from the literature above, there is a scarcity of reliable thermochemical data for N‐containing species. Although Wang et al. [36] recently evaluated the thermodynamic properties of 283 stable N‐containing species (excluding radicals) using high‐level quantum chemical methods; similar computations for species related to detailed pyrrole chemistry do not exist. This work aims to address this gap in thermochemical data of pyrrole pyrolysis and combustion models by computing the enthalpy of formation, entropy, and specific heat capacities across a range of temperatures. Key species relevant to JSR experiments by Pelucchi et al. [12] were considered for this purpose. After the evaluation of thermochemical data with high‐level ab initio methods, the effects of the revised data on overall species profile predictions were analyzed. The results in this work contribute to a more accurate and extensive thermochemical data set for pyrrole pyrolysis and combustion mechanism.

## 2 Methodology

This work re‐evaluated the thermodynamic parameters of key species in pyrrole chemistry. The effects of the new dataset on the performance of two models from the literature were also assessed with the new thermodynamic data. This way, the importance of thermodynamic parameters on pyrrole combustion chemistry was clarified. A combination of ab initio calculations and chemical kinetic simulations was implemented for pyrrole pyrolysis and combustion in this work.

### 2.1 Ab Initio Calculations

The relevant species of pyrrole combustion as listed in Pelucchi et al. [12], were considered as key species. The thermodynamic parameters of these key species were re‐evaluated in ORCA 6.1.1 [37, 38] that depends on the SHARK integral package [39]. For these calculations, the equilibrium geometry was optimized, and the frequencies were evaluated within the density functional theory (DFT) framework [40, 41]. For all radicals, the spin‐unrestricted computations were done. No molecular symmetry was imposed during the calculations. It has been demonstrated that the correction terms to the thermochemical properties arising from the internal rotations are insignificant in combustion chemical kinetic modeling context [42, 43]. Therefore, internal rotations were not considered for the small molecules studied in this work. For $O_{2}$, the triplet state has been used for the ground state. The PBE0 functional [44] together with Ahlrichs quadruple‐ζ basis set (def2‐QZVP) [45, 46, 47] level of theory was adopted for these computations. The entropy of formation at 298.15 K ($S298_{i}$) for species "i" was determined at this level. The total energy (ECCSD(T)) of the optimized structures was calculated at the coupled cluster singles doubles perturbative triples (CCSD(T)) level of theory. For the CCSD(T) calculation, the automatic basis set extrapolation (Extrapolate(3/4,def2)) with automatic generation of auxiliary basis sets (autoaux) [48] was used. With the Extrapolate(3/4,def2) option, ORCA 6.1.1 [37, 38] performs two‐point complete‐basis‐set (CBS) extrapolation of energy using Ahlrichs triple‐ζ (def2‐TZVP) and def2‐QZVP basis sets [45]. The input and output files from these calculations are available in the Supporting Information.

The corrected enthalpy ($H_{\mathrm{QM}@298K}$) at 298.15 K was calculated from the equation $H_{\mathrm{QM}@298K}$ = ECCSD(T) + $E_{\mathrm{ZPE}}$ + $E_{\mathrm{vib}@298K}$ + $E_{rot@298K}$ + $E_{\mathrm{tran}@298K}$ + $E_{\mathrm{therm}@298K}$, where $E_{\mathrm{ZPE}}$ is the zero point energy, $E_{\mathrm{vib}@298K}$ is the vibrational correction at 298.15 K, $E_{\mathrm{rot}@298K}$ is the rotational correction at 298.15 K, $E_{\mathrm{tran}@298K}$ is the translational correction at 298.15 K, and $E_{\mathrm{therm}@298K}$ is the thermal enthalpy correction at 298.15 K. Finally, the enthalpy of formation at 298.15 K ($H298_{i}$) for species "i' was determined with the atomization method [49]. It can be noted here that the reference standard formation enthalpies of the C, H, O, and N atoms were calculated from the same calculation method described above with naphthalene, hydrogen, oxygen, and nitrogen molecules, respectively. The enthalpy of formation of naphthalene at 298.15 K was considered to be 147.69 kJ/mol [50]. A sample calculation is provided in the Supporting Information.

### 2.2 Thermochemical Parameter Evaluation and Validation

After the ab initio calculations, coefficients of the NASA polynomials were generated in the 300–5000 K range using the FITDAT utility in ANSYS CHEMKIN‐PRO software [51]. The FITDAT output files are provided as Supporting Information to show the relative error values and other relevant parameters during data fitting. These NASA polynomial coefficients were added to the thermodynamic data of two models from the literature, i.e., Pelucchi et al. [12] and Konnov et al. [52]. The updated thermodynamic dataset, along with a comparison of its thermochemical parameters with those reported by Pelucchi et al. [12] is available in the Supporting Information. Finally, the changes in the performances of Pelucchi et al. [12] and Konnov et al. [52] models due to the updated thermodynamic dataset were assessed. For this purpose, the experimental data from an atmospheric pressure JSR in Nancy [12] was used. To simulate this reactor, the Perfectly Stirred Reactor (PSR) model in ANSYS CHEMKIN‐PRO [51] was utilized.

In the next section, the thermodynamic parameters computed in this work are compared with the values from the literature. Then, the effects of the revised thermodynamic parameters on the prediction of the experimental data [12] are discussed.

## 3 Results and Discussion

### 3.1 Thermodynamic Parameters

Table 1 shows the comparison of H298 and S298 computed in this work and in the literature. It can be noted here that the data from the literature in Table 1 have been used by both Pelucchi et al. [12] and Konnov et al. [52]. Additionally, the literature data also includes recently published values from Wang et al. [36] and Konnov et al. [54]. It can be seen from the table that H298 computed in this work deviates from the corresponding value in Pelucchi et al. [12] by around 44.18 kcal/mole for allyloxy cyanide ($C_{4}H_{4}\text{NO}$) because of structural differences. Specifically, Pelucchi et al. [12] consider $C_{4}H_{4}\text{NO}$ has a structure O(.)─CH═CH─‐CH_2_─C#N, while in the present work the calculations have been performed for O═CH─CH(.)─CH_2_─C#N radical. The structure of the $C_{4}H_{4}\text{NO}$ radical molecule in the present work can be found in the Supporting Information. For *cis*‐formimidoyl and cyanomethylidyne, the deviation from literature data [12] is around 12.2 and 10.37 kcal/mole, respectively. However, for both *cis*‐formimidoyl and cyanomethylidyne, the calculated H298 values in this work agree well with the Burcat [53] database. Similarly, the deviation is around 8.37 and 9.42 kcal/mole for the cyano‐propen‐4yl radical and carbonyl cyanide, respectively, from the literature [12, 55]. For cyano‐propen‐2yl and propionitrile primary radicals, the observed deviations are 7.89 and 5.67 kcal/mole, respectively, with respect to the literature [55, 57]. All other H298 values computed in this work have been found to be close to the literature data. On the other hand, for S298, cyano‐propen‐4yl radical and carbonyl cyanide show a deviation of 5.9 and 5.68 cal/mole‐K, respectively. All other S298 values computed in this work are within 4 cal/mole‐K of the literature data. It can be noted here that the recently published data from Wang et al. [36] and Konnov et al. [54] are close to the values computed in the present work for both H298 and S298.

**TABLE 1:** Enthalpy and entropy of formation values computed in this work and compared against the literature. Units: H298 [kcal/mole] and S298 [cal/mole‐K]. Names of the compounds are according to the mechanism files used in this work.

Compound	Composition				Name	Present work		Literature data		References
	C	H	N	O		H298	S298	H298	S298	
2‐Butynedinitrile	4	0	2	0	C_4_N_2_	128.53	68.09	126.5	69.4	[53]
Acetonitrile	2	3	1	0	CH_3_CN	17.61	60.10	17.7	58.1	[53]
Acrylonitrile	3	3	1	0	CH_2_CHCN	44.94	65.17	44	62.9	[53]
								43.99	65.21	[54]
Acrylonitrile radical	3	2	1	0	CHCHCN	107.19	66.38	105.8	65	[53]
Allenic imine	4	5	1	0	HNCPROP	68.82	73.62	68.7	74.6	[55]
Allyl cyanide	4	5	1	0	A‐C_3_H_5_CN	39.89	72.95	38	74.4	[56]
								40.09	75.77	[36]
Allyloxy cyanide	4	4	1	1	C_4_H_4_NO	30.22	79.56	74.4	80.6	[12]
Butanedinitrile	4	4	2	0	C_4_H_4_N_2_	49.93	77.31	50.1	77.7	[53]
Butanedinitrile radical	4	3	2	0	C_4_H_3_N_2_	93.10	79.39	91.2	80.5	[57]
Carbonyl cyanide	2	0	1	1	NCCO	59.82	57.52	50.4	63.2	[12]
*cis*‐Crotonitrile	4	5	1	0	C‐C_3_H_5_CN	35.18	73.18	32	71.6	[56]
								35.81	76.2	[36]
Cyano allyl radical	4	4	1	0	A‐C_3_H_4_CN	70.02	72.92	68.2	73.6	[55]
Cyano methyl radical	2	2	1	0	CH_2_CN	62.43	59.30	62.9	59.9	[12]
								61.61	61.14	[53]
Cyano‐propen‐2yl radical	4	4	1	0	C_3_H_4_CN	92.79	75.63	84.9	72.1	[55]
Cyano‐propen‐4yl radical	4	4	1	0	C‐C3H_4_CN	99.87	74.19	91.5	80.1	[55]
Cyano radical	1	0	1	0	CN	106.70	48.36	104.85	48.43	[53]
Cyanoacetylene	3	1	1	0	C_3_HN	90.15	59.47	88.05	59.26	[53]
								89.97	59.71	[36]
Cyanomethylene radical	2	1	1	0	HCCN	115.08	55.77	113.9	59.2	[53]
Cyanomethylidyne	2	0	1	0	C_2_N	163.03	53.70	173.4	57.1	[12]
								162.3	56.67	[53]
*cis*‐Formimidoyl	1	2	1	0	CH_2_N	69.20	54.70	57	53.6	[12]
								69.5	54.8	[53]
Formyl cyanide	2	1	1	1	OCHCN	11.67	64.70	11.5	64.8	[12]
Fumaronitrile	4	2	2	0	C_4_H_2_N_2_	81.28	73.62	80.9	71.6	[57]
								81.36	71.45	[36]
Hydrogen cyanide	1	1	1	0	HCN	31.02	48.06	31.02	48.23	[53]
Hydroxy acetonitrile radical	2	2	1	1	OCH_2_CN	42.49	67.05	43.9	67.2	[12]
Isocyanate radical	1	0	1	1	NCO	29.89	53.40	30.6	55.5	[53]
Isocyanic acid	1	1	1	1	HNCO	−29.02	56.92	−28.35	56.94	[53]
Propionitrile	3	5	1	0	C_2_H_5_CN	12.86	68.02	12.7	68.1	[53]
Propionitrile primary radical	3	4	1	0	CH_2_CH_2_CN	63.87	67.85	58.2	68.5	[57]
Propionitrile secondary radical	3	4	1	0	CH_3_CHCN	53.98	70.37	54.2	69.5	[57]
								53.22	69.19	[53]
Pyrrole	4	5	1	0	C_4_H_5_N	25.26	65.71	25.3	64.6	[12]
								26.23	66.14	[36]
								25.60	66.02	[54]
Pyrrolenine	4	5	1	0	PYRLNE	39.31	65.97	40.6	66.1	[12]
Pyrrolyl radical	4	4	1	0	PYRLYL	68.74	66.88	69.3	65.7	[12]

The isobaric specific heats of the species in Table 1 at a temperature range of 300–3000 K computed in the present work are also compared with those from Pelucchi et al. [12] in the Supporting Information. The observable differences in the specific heats may be attributed to the differences in thermodynamic parameters between the present work and the literature data. It can be noted here that the thermochemical dataset of Pelucchi et al. [12] is also a compilation from a wide range of literature sources.

### 3.2 Changes in Model Performance

**FIGURE 1:**
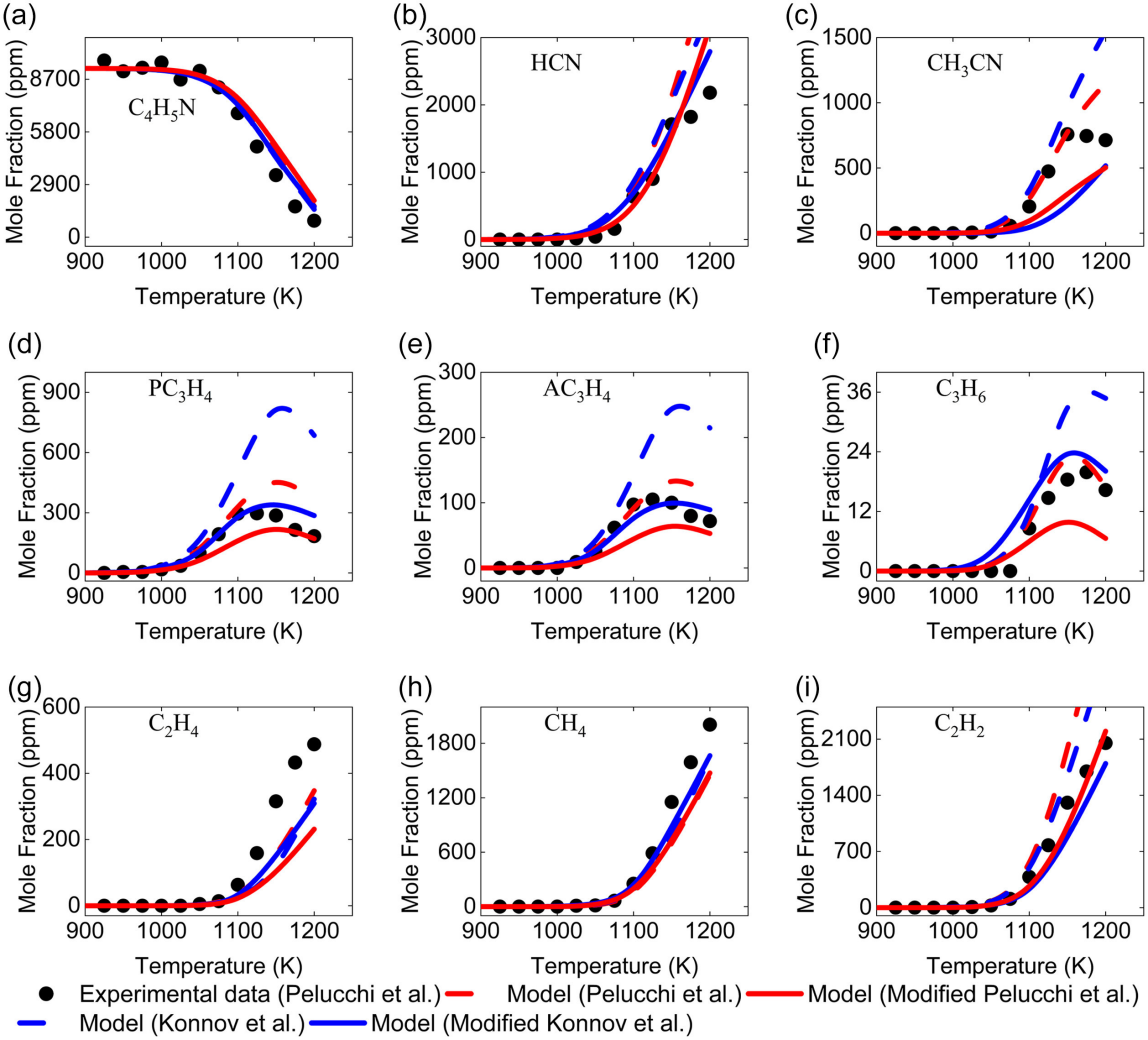
Validation against experimental data from Pelucchi et al. [12] for pyrolysis of pyrrole (∼1 mol% in helium) at atmospheric pressure and residence time of 2 s. Panels (a)−(i) present mole fractions of C_4_H_5_N, HCN, CH_3_CN, PC_3_H_4_, AC_3_H_4_, C_3_H_6_, C_2_H_4_, CH_4_, and C_2_H_2_, respectively.

Next, the effects of changes in the thermodynamic data of species mentioned in Table 1 on the overall model [12, 52] performances have been assessed. Figure 1a–i shows the comparison of model [12, 52] predictions with experimental data [12] for the pyrolysis of pyrrole. It can be observed from Figure 1a that the changes made in this work do not have significant influence on the prediction of pyrrole mole fraction for both mechanisms [12, 52]. For HCN in Figure 1b, marginal improvement in the performance of both models can be observed after the thermodynamic parameter update. In contrast, Figure 1c shows that the thermodynamic parameter update does not always improve the model prediction. Figure 1d–f shows that the advancement in accuracy of the thermodynamic data improves the predictions of the mole fraction of ${\text{PC}}_{3}H_{4}$, ${\text{AC}}_{3}H_{4}$, and $C_{3}H_{6}$ for the Pelucchi et al. [12] model. For $C_{2}H_{4}$ and ${\text{CH}}_{4}$, as shown in Figure 1g,h, the changes in the thermodynamic data do not change the model performances significantly. However, the prediction accuracy of both models [12, 52] show improvement for $C_{2}H_{2}$ mole fractions in Figure 1i.

**FIGURE 2:**
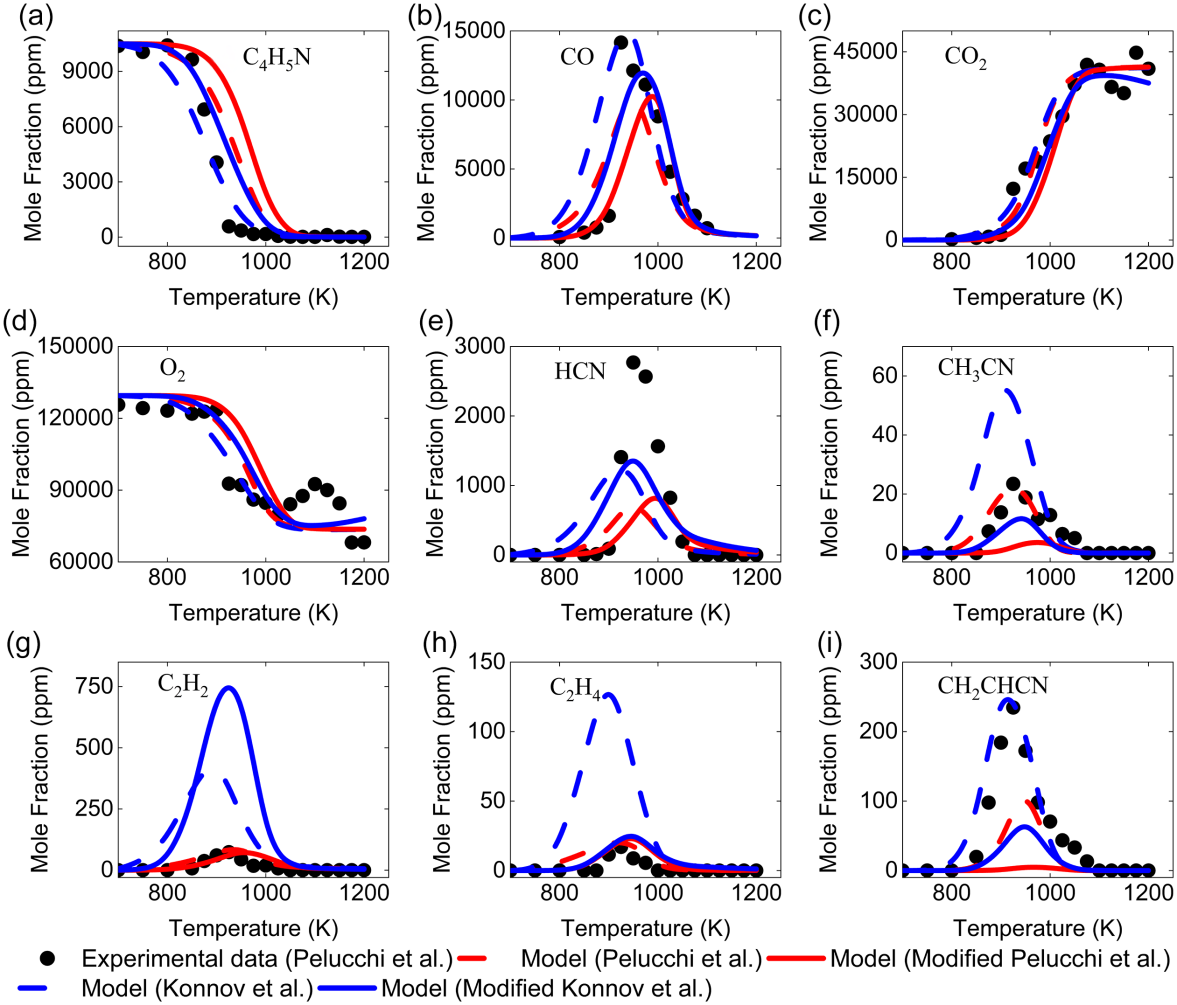
Validation against experimental data on pyrrole combustion from Pelucchi et al. [12] at atmospheric pressure, φ = 0.5, and residence time of 2 s. Panels (a)−(i) present mole fractions of C_4_H_5_N, CO, CO_2_, O_2_, HCN, CH_3_CN, C_2_H_2_, C_2_H_4_, and CH_2_CHCN, respectively.

Figure 2a–i shows the comparison of the model [12, 52] performances with experimental data [12] for pyrrole oxidation at equivalence ratio φ = 0.5. It can be observed from Figure 2a that the prediction of pyrrole mole fraction improves for the Konnov et al. [52] model after the changes in thermodynamic parameters. However, for the Pelucchi et al. [12] mechanism, the trend is marginally opposite. For CO in Figure 2b, the updated Pelucchi et al. model [12] performs slightly better than the Konnov et al. [52] model. It is not clear if the modified thermodynamic parameters improve the model performance in Figure 2c,d due to the scatter in experimental data. The peak HCN mole fraction is underpredicted by both models [12, 52], as shown in Figure 2e. However, marginal improvements in predicting the HCN mole fraction can also be observed in both models [12, 52] after the modifications in this work. For ${\text{CH}}_{3}\text{CN}$ in Figure 2f, the Konnov et al. [52] model performs significantly better with the new thermodynamic data. On the other hand, for $C_{2}H_{2}$ in Figure 2g, the modified Pelucchi et al. [12] mechanism performs significantly better than the Konnov et al. [52] model. For $C_{2}H_{4}$ in Figure 2h, the Konnov et al. [52] model shows significant improvement after the modifications in the thermodynamic rata. However, the Pelucchi et al. [12] mechanism performs significantly better in predicting the mole fraction of $C_{2}H_{4}$. Finally, for ${\text{CH}}_{2}\text{CHCN}$ as in Figure 2i, both models [12, 52] show significant inaccuracies after the modifications.

**FIGURE 3:**
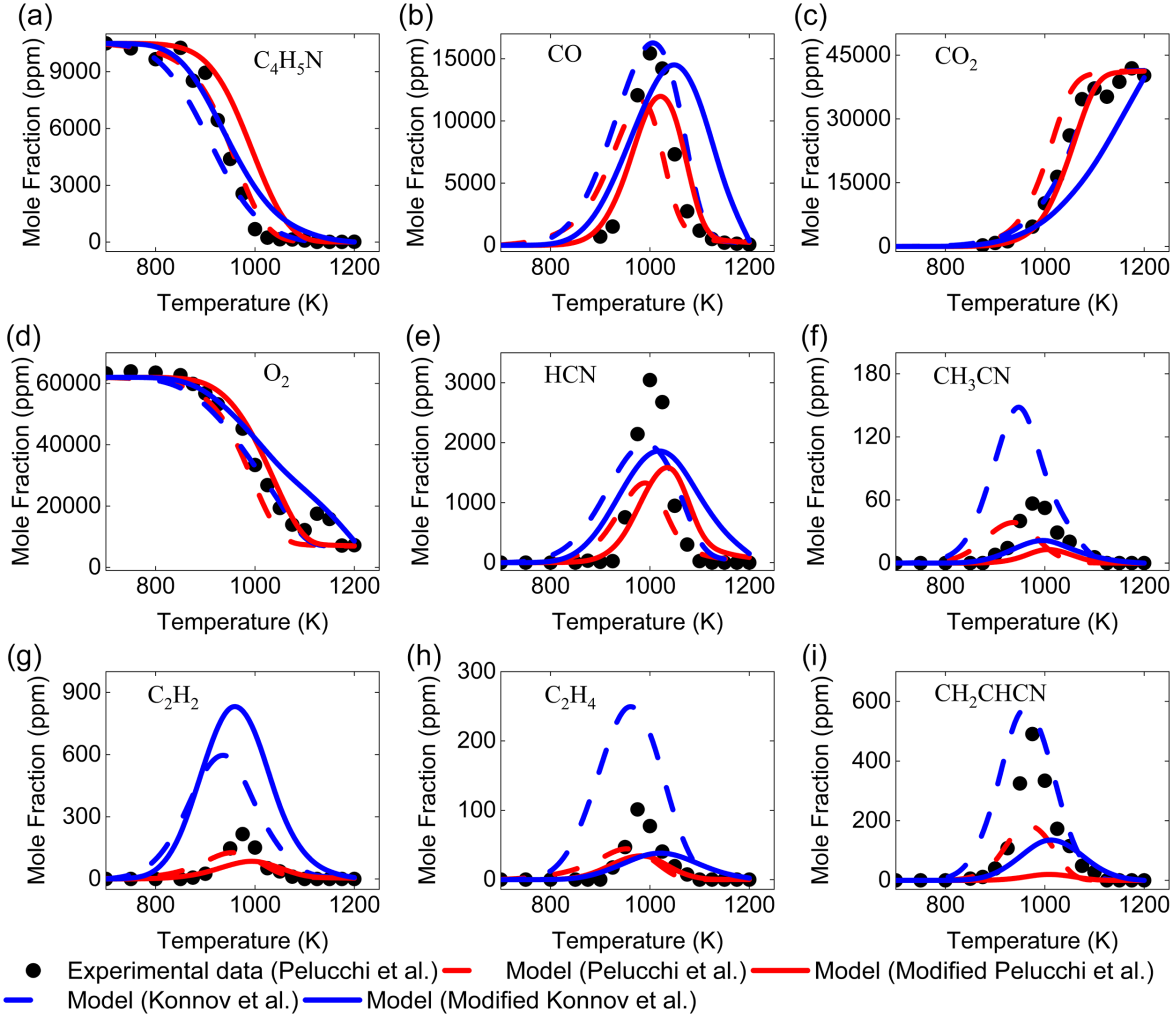
Validation against experimental data on pyrrole combustion from Pelucchi et al. [12] at atmospheric pressure, φ = 1, and residence time of 2 s. Panels (a)−(i) present mole fractions of C_4_H_5_N, CO, CO_2_, O_2_, HCN, CH_3_CN, C_2_H_2_, C_2_H_4_, and CH_2_CHCN, respectively.

Figure 3a–i shows the comparison of the model [12, 52] performances with experimental data [12] for pyrrole oxidation at φ = 1. Figure 3a shows a modest improvement in the prediction of pyrrole consumption after the changes made in this work for the Konnov et al. model [52] at temperatures below 1000 K. On the other hand, for CO in Figure 3b, the modifications in thermodynamic parameters improve the predictions by Pelucchi et al. [12] model. However, an opposite trend can be observed for Konnov et al. [52] model is shown in Figure 3b. In Figure 3c,d, the modifications done in the present work improve the predictions of Pelucchi et al. [12] model marginally. Similar to Figure 2e, Figure 3e also shows that the peak HCN mole fraction is underpredicted by both models [12, 52]. For ${\text{CH}}_{3}\text{CN}$ in Figure 3f, the Konnov et al. [52] model performs slightly better than the Pelucchi et al. [12] model with the new thermodynamic data. The Konnov et al. [52] model also improves significantly after the thermodynamic parameter modifications. However, in Figure 3g‍–‍i, the changes made in the present work do not bring about clear improvements in predictions by both models [12, 52].

**FIGURE 4:**
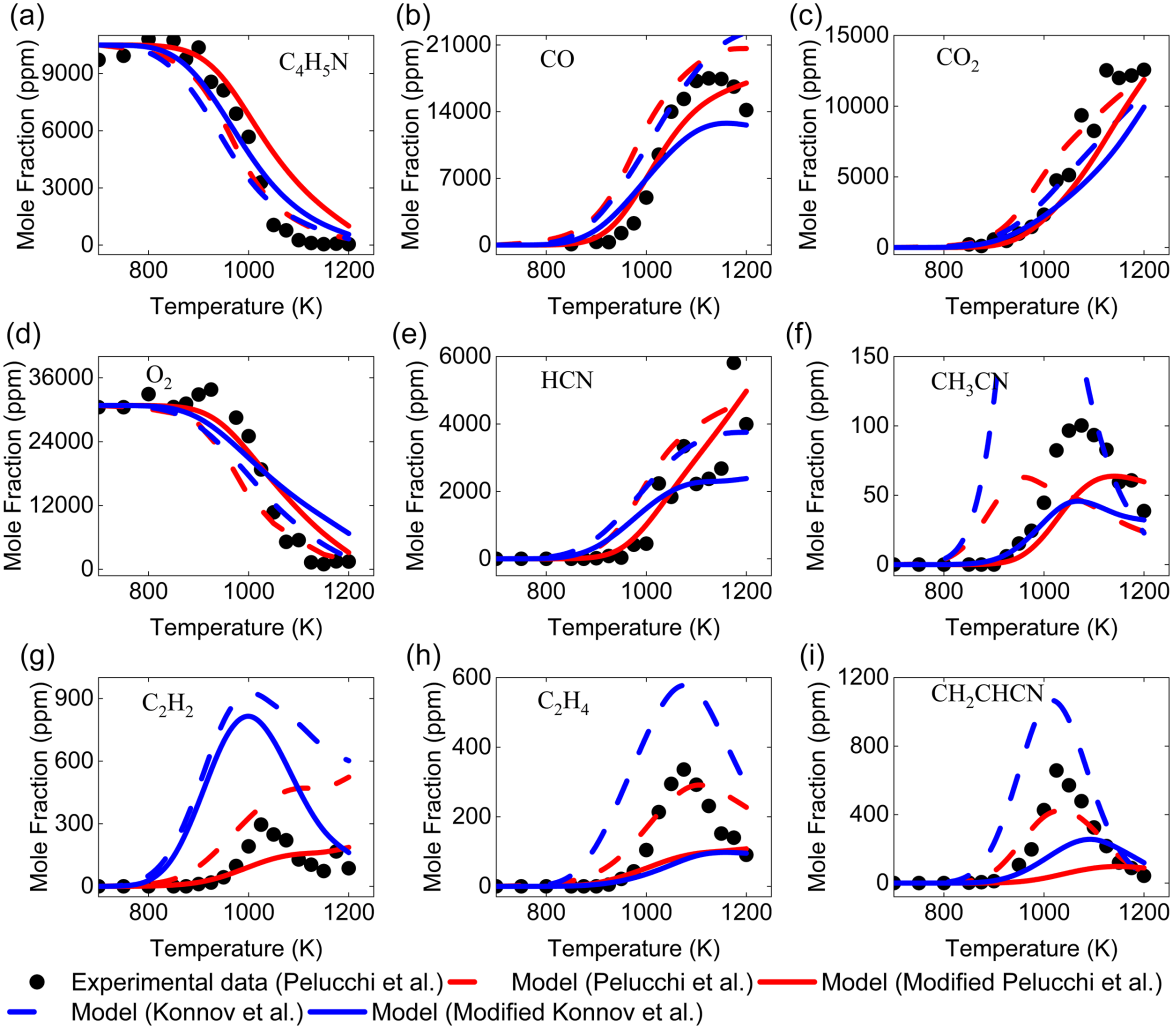
Validation against experimental data on pyrrole combustion from Pelucchi et al. [12] at atmospheric pressure, φ = 2, and residence time of 2 s. Panels (a)−(i) present mole fractions of C_4_H_5_N, CO, CO_2_, O_2_, HCN, CH_3_CN, C_2_H_2_, C_2_H_4_, and CH_2_CHCN, respectively.

Figure 4a–i shows the comparison of model [12, 52] performances with experimental data [12] for pyrrole oxidation at φ = 2. Similar to the abovementioned pyrolysis and φ = 0.5–1 cases, the changes in thermodynamic parameters in the present work bring about noticeable changes in model performances for all species in Figure 4. As seen from Figure 4a, there is a modest improvement in the prediction of pyrrole consumption after the changes made in this work for both mechanisms [12, 52] at temperatures below 1000 K. In Figure 4b–e, the modified models predict the experimental data quite acceptably. However, the improvements in predictions are not clear, as shown in Figure 4f–i.

### 3.3 Sensitivity Analysis

**FIGURE 5:**
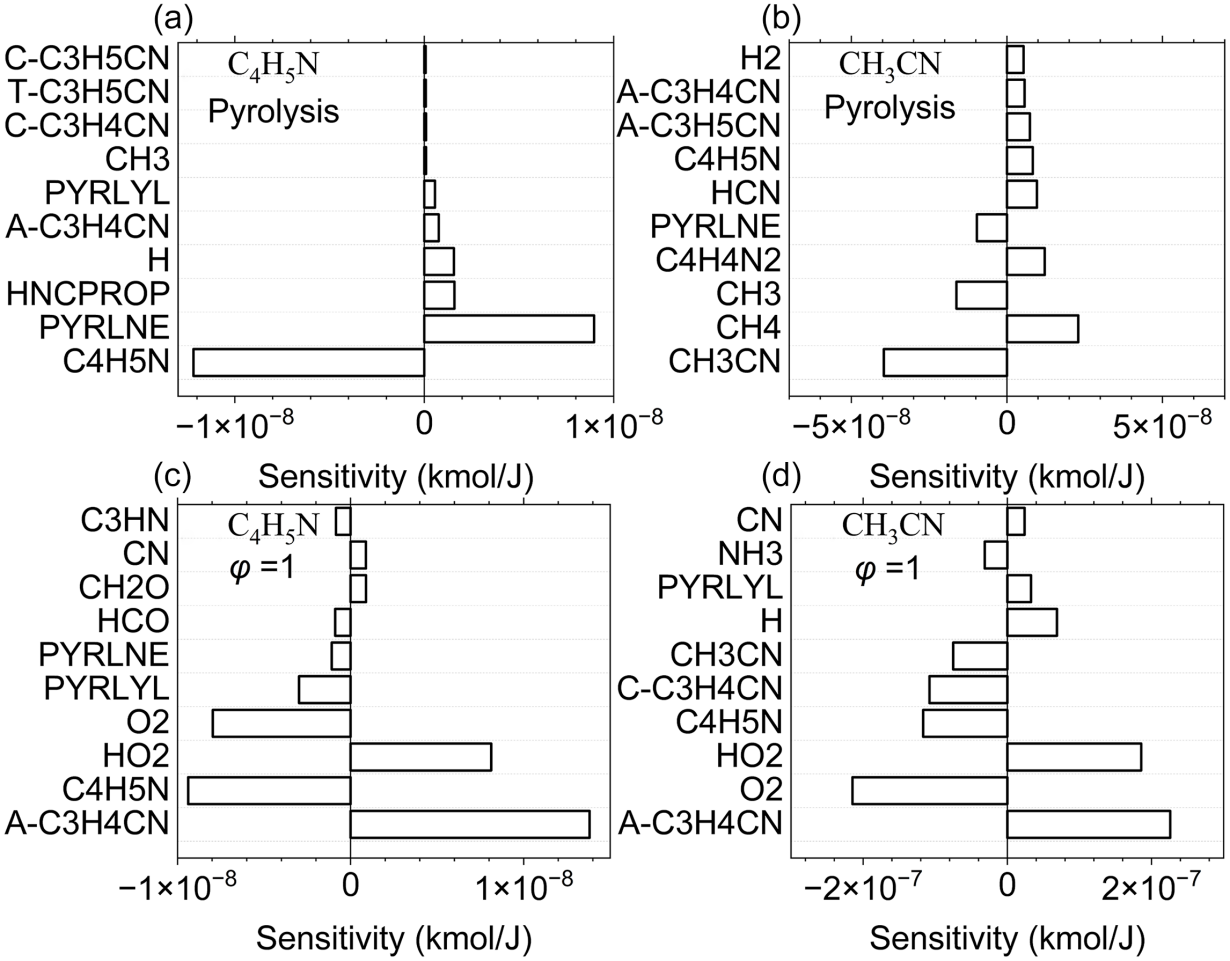
Heat of formation sensitivity analysis with the Pelucchi et al. [12]. model on the mole fractions of (a) pyrrole at 1100 K, (b) acetonitrile at 1200 K during pyrolysis and (c) pyrrole at 900 K, and (d) acetonitrile at 1000 K during combustion at φ = 1.

Next, the heat of formation sensitivity analysis with Pelucchi et al. [12] model on species mole fractions is shown in Figure 5a–d to analyze the discrepancies in mechanism validation results (Figure 1–4). For this purpose, the chosen species are pyrrole ($C_{4}H_{5}N$), for which the changes made in Table 1 mostly improve the predictions in Figures 1–4, and acetonitrile (${\text{CH}}_{3}\text{CN}$), for which the changes made in Table 1 worsen the predictions in Figures 1–4. This enthalpy of formation sensitivity analysis has been done in Cantera 2.6.0 [58] with the add_sensitivity_species_enthalpy (k) command. Here, the sensitivity coefficient ($S_{k}$) for species k is defined as

(1) $$S_{k}=\frac{1}{X_{\mathrm{target}}}\frac{\partial X_{\mathrm{target}}}{\partial HoF_{k}}$$

where ${\mathrm{HoF}}_{k}$ is the enthalpy of formation of species k and $X_{\mathrm{target}}$ is the mole fraction of either $C_{4}H_{5}N$ or ${\text{CH}}_{3}\text{CN}$. A similar sensitivity analysis can be found in the recent work of Bhattacharya et al. [59].

It can be seen from Figure 5a that the pyrrole mole fraction during the pyrolysis at 1100 K is primarily influenced by the enthalpy of formation of pyrrole and pyrrolenine. As shown in Table 1, the H298 values of both pyrrole and pyrrolenine computed in this work are quite close to the literature data [12, 36, 54]. Therefore, Figure 5a explains the fact that the modified models in this work predict $C_{4}H_{5}N$ mole fractions in Figure 1a that are quite close to the original models [12, 52]. However, for acetonitrile (${\text{CH}}_{3}\text{CN}$) in Figure 5b, the highest sensitivity is shown for acetonitrile itself. As shown in Table 1, the H298 of acetonitrile computed in this work is almost the same as the literature data [53]. Additionally, the difference between S298 computed in this work and literature data [53] for acetonitrile is also around 2 cal/mole‐K. For butanedinitrile ($C_{4}H_{4}N_{2}$), the computed values of H298 and S298 in the present work are also close to the literature data in Table 1. Therefore, it may be concluded that the differences observed in Figure 1c between the experimental data [12] and modified model [12, 52] predictions do not directly originate from the H298‐sensitive species. Rather, these differences originate from changes in thermodynamic data of species that affect the mole fraction predictions indirectly through chemical reactions.

For the combustion condition at φ = 1 for pyrrole in Figure 5c, the highly sensitive species are cyano allyl radical (A‐$C_{3}H_{4}\text{CN}$) and pyrrole itself. On the other hand, for ${\text{CH}}_{3}\text{CN}$ in Figure 5d, the highest sensitivity is also observed for A‐$C_{3}H_{4}\text{CN}$. Furthermore, cyano‐propen‐4yl radical (C‐$C_{3}H_{4}\text{CN}$) also shows high sensitivity. It can be seen from Table 1 that the H298 value computed in this work differs from the literature [55] by around 8.37 kcal/mol for C‐$C_{3}H_{4}\text{CN}$. For the S298 of C‐$C_{3}H_{4}\text{CN}$, the difference is around 6 cal/mole‐K. Therefore, the differences in predicted mole fractions of ${\text{CH}}_{3}\text{CN}$ between the modified and original models in Figures 2–4 may be attributed to the differences in presently computed H298 and S298 values of C‐$C_{3}H_{4}\text{CN}$ with respect to the literature [55] data. Additionally, it may be noted that the chemical effects originating from the changes in thermodynamic data may also affect the mole fraction predictions.

It is a known fact that thermochemistry is crucial for accurate chemical kinetic modeling. As mentioned earlier, vom Lehn et al. [24, 25, 26] have done some extensive work on the effects of thermochemical parameters on the combustion characteristics of hydrocarbon fuels. Similarly, Bugler et al. [60] have shown how the nitrogenated species profiles depend on the thermochemical parameters at gas turbine‐relevant conditions, i.e., atmospheric pressure and high temperature. In this regard, the present work demonstrates that the influence of thermodynamic parameters on pyrrole combustion chemistry is more severe than that on hydrocarbon fuels in vom Lehn et al. [24, 25, 26] and Bugler et al. [60] The results in this work call for more rigorous efforts in chemical kinetic modeling of cyclic species with N‐heteroatoms, taking both reaction and thermochemical parameters into account.

## 4 Conclusion

This work presents high‐level quantum chemical calculations of thermochemical properties for species relevant to the pyrolysis and combustion of pyrrole. The models from Konnov et al. [52] and Pelucchi et al. [12] were updated with the revised thermochemical parameters, and the performances were compared against the experimental data [12]. Through systematic chemical kinetic validations, key insights were gained into the influence of thermochemistry on the pyrolysis and combustion of pyrrole.

It was observed that the H298 and S298 values of pyrrole, *cis*‐crotonitrile, allyl cyanide, acrylonitrile, cyanoacetylene, and fumaronitrile computed in this work agree well with the recently published values in Wang et al. [36] and Konnov et al. [54]. Furthermore, most of the thermochemical data computed in this work match those from Pelucchi et al. [12]. However, the present work also computed some H298 and S298 values that are significantly different from those of Pelucchi et al. [12]. For example, the H298 of allyloxy cyanide computed in this work differs from that of Pelucchi et al. [12] by around 44.18 kcal/mole. Additionally, the H298 values of cyanomethylidyne, cyano‐propen‐4yl radical, carbonyl cyanide, cyano‐propen‐2yl radical, and propionitrile primary radical computed in this work are significantly different from literature. The S298 values of cyano‐propen‐4yl radical and carbonyl cyanide also show considerable deviation from literature data.

After the changes to the thermodynamic parameters of Konnov et al. [52] and Pelucchi et al. [12] models, there were noticeable changes in validation results against the JSR data from Pelucchi et al. [12]. Pyrrole concentration prediction seemed to have either improved or remained nearly unchanged at most experimental conditions. However, due to the combined effect of changes in the thermodynamic properties and reaction rates, the predictions of the mole fraction of acetonitrile with the modified thermodynamic parameters are also considerably different from the original study by Konnov et al. [52] and Pelucchi et al. [12] models. Similar observations have also been made for other species at pyrolysis and φ = 0.5–1 conditions. These facts indicate that the chemical reaction parameters need to be adjusted after the thermodynamic update. However, reaction parameters are out of the scope of the present work.

This work highlights the need for more attention to the thermodynamic data in chemical kinetic modeling of pyrrole combustion. Future work should focus on optimizing the reaction rate parameters based on the revised thermodynamic data from this work. Furthermore, the methodology proposed in this work can be applied to other fuel molecules to improve the accuracy of thermodynamic parameters. In future, internal rotations may be considered for molecules relevant to pyrrole combustion chemistry.

## Funding

This study was supported by Academy of Finland (346918) and Knut och Alice Wallenbergs Stiftelse (KAW2019.0084COCALD).

## Conflicts of Interest

The authors declare no conflicts of interest.

## Supporting information

Supplementary Material

## Data Availability

The data that supports the findings of this study are available in the Supporting Information of this article.
