# Supplementary material for: The Importance of Thermodynamic Data for Pyrrole Pyrolysis and Combustion Chemistry
Source: Chemphyschem. 2026 Sep 20;27(18):e70563. doi: 10.1002/cphc.70563 (PMC13590417; doi:10.1002/cphc.70563)
Supplement: Supplementary file 1 [file CPHC-27-e70563-s001.zip › supplementary_materialPyrrolePaper/sample_calculation.docx]

# Sample Calculation for Determining the H298 of Pyrrole

According to the atomization method^­­^ [Combustion and Flame 2023, 252, 112742], the H298 of a molecule with composition C_x_H_y_O_z_ is given by,

$$H298(C_{x}H_{y}O_{z})= H_{QM@298K}(C_{x}H_{y}O_{z})-x H_{QM@298K}(Graphite)-\frac{y}{2}H_{QM@298K}(H_{2})-\frac{z}{2}H_{QM@298K}(O_{2})$$

... equation 1

Where,

$$H_{QM@298K}= ECCSD(T) + E_{\mathrm{ZPE}} +E_{vib@298K} +E_{rot@298K}+ E_{tran@298K} + E_{therm@298K}$$

Equation 1 has been used to determine “$H_{QM}(Graphite)$” from $H298(Naphthalene)= 147.69 kJ/mol$[Active Thermochemical Tables (ATcT) ver. 1.176].

Therefore, the expression for $H298(\text{Pyrrole)}$ according to equation 1 is,

$$H298(\text{Pyrrole)}=\left( H_{QM@298K}(\text{Pyrrole)}-\text{4}\times\frac{\frac{147.69}{2625.5}-H_{QM@298K}(\text{Naphthalene)}+4\times H_{QM@298K}(H_{2})}{-10}-\frac{\text{1}}{2}\times H_{QM@298K}(N_{2})-\frac{\text{5}}{2}\times H_{QM@298K}(H_{2})-\frac{\text{0}}{2}\text{×}H_{QM@298K}(O_{2}) \right)\times627.503 [kcal/mol]$$

... equation 2

Where, $H_{QM@298K}$is given is Hartree (Eh).

The following is a python script that calculates $H298(\text{Pyrrole)}$ according to equation 2.

**Python code to calculate H298 of Pyrrole:**

#Naphthalene values

sp_energy = -385.334290003264 # $ECCSD(T)$

total_corr = 0.15482516 #$E_{\mathrm{ZPE}} +E_{vib@298K} +E_{rot@298K}+ E_{tran@298K}$

thermal_enthalpy_corr = 0.00094373 #$E_{therm@298K}$

Naphthalene = sp_energy+total_corr+thermal_enthalpy_corr

#Hydrogen values

sp_energy = -1.17464268189 # $ECCSD(T)$

total_corr = 0.01239917 #$E_{\mathrm{ZPE}} +E_{vib@298K} +E_{rot@298K}+ E_{tran@298K}$

thermal_enthalpy_corr = 0.00094373 #$E_{therm@298K}$

Hydrogen = sp_energy+total_corr+thermal_enthalpy_corr

#Nitrogen values

sp_energy = -109.422054695575 # $ECCSD(T)$

total_corr = 0.00800897 #$E_{\mathrm{ZPE}} +E_{vib@298K} +E_{rot@298K}+ E_{tran@298K}$

thermal_enthalpy_corr = 0.00094373 #$E_{therm@298K}$

Nitrogen = sp_energy+total_corr+thermal_enthalpy_corr

#Oxygen values

sp_energy = -150.199890795515 # $ECCSD(T)$

total_corr = 0.00626235 #$E_{\mathrm{ZPE}} +E_{vib@298K} +E_{rot@298K}+ E_{tran@298K}$

thermal_enthalpy_corr = 0.00094373 #$E_{therm@298K}$

Oxygen = sp_energy+total_corr+thermal_enthalpy_corr

#Pyrrole values

no_carbon = 4

no_hydrogen = 5

no_nitrogen = 1

no_oxygen = 0

sp_energy = -209.893338507725 # $ECCSD(T)$

total_corr = 0.08702098 #$E_{\mathrm{ZPE}} +E_{vib@298K} +E_{rot@298K}+ E_{tran@298K}$

thermal_enthalpy_corr = 0.00094373 #$E_{therm@298K}$

Pyrrole = sp_energy+total_corr+thermal_enthalpy_corr

#Enthalpy of formation calculation

H298 = (((Pyrrole)

- no_carbon * ((147.69/2625.5 - Naphthalene + 4 * Hydrogen) / -10.0)

- (no_nitrogen / 2.0) * Nitrogen

- (no_hydrogen / 2.0) * Hydrogen

- (no_oxygen / 2.0) * Oxygen))*627.503

print (H298)
